# Neurocognitive and adaptive phenotypes in adolescent inpatients with mood disorders: an exploratory study on disruptive mood dysregulation disorder in the framework of depressive disorders

**DOI:** 10.3389/fpsyt.2023.1253589

**Published:** 2023-11-27

**Authors:** Massimo Apicella, Marcella Caterina Pisa, Roberto Averna, Milena Labonia, Maria Pontillo, Stefano Vicari

**Affiliations:** ^1^Child Neuropsychiatry Unit, Department of Neuroscience, Bambino Gesù Children’s Hospital, IRCCS, Rome, Italy; ^2^Department of Life Sciences and Public Health, Catholic University, Rome, Italy

**Keywords:** disruptive mood dysregulation disorder, depression, adolescent, neurodevelopment, irritability, adaptive abilities, intelligence, neuropsychology

## Abstract

**Introduction:**

Few studies on adolescents have investigated intelligence quotient (IQ) in mood disorders. Evidence on Disruptive Mood Dysregulation Disorder (DMDD), a controversial entity among depressive disorders, is more limited.

**Materials and methods:**

We performed an exploratory study on adolescent inpatients with unipolar mood disorders to test specific impairment in cognitive and adaptive profile. We also considered common psychopathological comorbidities. We retrospectively collected data on inpatients with a diagnosis of major depressive disorder (MDD), DMDD or Depressive Disorder – Not Otherwise Specified (DD-NOS) evaluated with Wechsler Scales of Intelligence, Adaptive Behavior Assessment System (ABAS-II), and Children’s Global Assessment Scale (C-GAS).

**Results:**

Out of 198 inpatients (85.9% females), 33.3% had MDD, 60.1% DD-NOS and 6.6% DMDD. DMDD patients had higher rates of ADHD (15.4%) and learning disorders (LD, 23.1%), a lower mean IQ (87.8 ± 10.7; *p* = 0.001) and ABAS-II scores (general composite 68.8 ± 16.8; *p* = 0.002) than other groups. In linear regression analysis, DMDD retained a significant correlation with lower IQ and adaptive abilities when controlling for sex, and comorbidities. Among comorbidities, LD correlated with lower perceptual reasoning and IQ, and ADHD with lower conceptual adaptive abilities. In all diagnosis groups, working memory and processing speed were lower than verbal comprehension and perceptual reasoning.

**Discussion:**

While impairment in working memory and processing speed is a non-specific correlate of active mood disorder, DMDD is burdened by lower general intelligence and adaptive abilities and higher rate of neurodevelopmental comorbidities. Lower IQ in the normal range is a correlate of DMDD among variables examined, not explained by the effect of neurodevelopmental comorbidities. These findings are discussed with regards to possible implications for the consideration of DMDD as a bridge condition between neurodevelopmental disorders and mood disorders.

## 1 Introduction

Mood disorders in adolescents are common and increasing in prevalence in recent times (1, 2). Unipolar depression is a particularly common and potentially disabling condition in adolescence (3), while bipolar disorder is rarer, but particularly severe (4). Chronic irritability is a common reason for a child and adolescent psychiatric consultation (5) and requires a careful psychodiagnostics evaluation. In DSM-5 (6), it appears among the diagnostic criteria of different non affective disorders such as Oppositional Defiant Disorder (ODD) and intermittent explosive disorder, among others (7). In children and adolescents, it is a main diagnostic feature for major depression. At this age, however, severe irritability is also the most common presentation of mania (8). Leibenluft et al. (9) described “broad phenotype” of pediatric bipolar disorder as characterized by chronic severe irritability. Following studies, however, clarified that children and adolescents with chronic irritability uncommonly progress to adult bipolar phenotypes (10) and more commonly develop major depressive disorder (MDD) and anxiety disorders (11). Furthermore, patients with severe mood dysregulation (SMD), an operationalization of severe chronic disability before DSM-5, rarely have family history of bipolar disorder (12). DSM-5, therefore, introduced the diagnosis of Disruptive Mood Dysregulation Disorder (DMDD) among depressive and related disorders to avoid overdiagnosis of pediatric bipolar disorder. This diagnosis and its placement among depressive and related disorders, however, has been criticized by many authors. Some remark the difficulty to distinguish DMDD from bipolar disorder in clinical settings (13), others comment on the difficulty to distinguish it from ODD (14) and neurodevelopmental disorders (15, 16). Some criticize the conceptualization of DMDD as a unique and separate disorder (17, 18), underlining possible overlap in neurobiology (19) and trajectory (16) between DMDD and attention deficit hyperactivity disorder (ADHD) subtypes. One study has recently evaluated DMDD in the framework of depressive and related disorders, by comparing clinical correlates, natural course, and vulnerability factors of inpatients with MDD, persistent depressive disorder and DMDD (20). Vulnerability factors were comparable among groups, but DMDD was characterized by more externalizing and trauma-related disorders, peer-relationship and school difficulties in comparison to MDD and persistent depressive disorder.

Cognitive domain is underinvestigated in mood disorders of adolescents in comparison to adults, and evidence on DMDD is particularly limited. A cognitive symptoms cluster is relevant in depression (21–23) and mania (24) and cognitive impairment may help characterize mood disorders and understand neural substrates involved in their pathogenesis (25).

There is some evidence that a lower Intelligence Quotient (IQ) is a nonspecific correlate across all psychiatric diagnoses (26, 27). With regard to studies on adults, IQ and working memory index (WMI) are impaired during unipolar depression (28) and cognitive impairment correlates with the severity of depressive symptoms in both unipolar and bipolar depression (29). Adults with bipolar disorder show a grade of impairment with lower inhibitory control, selective attention and verbal and visual memory performances even when euthymic (30). Premorbid IQ of patients with bipolar disorder, however, is not lower than the general population (31, 32).

Studies on cognition in adolescents with mood disorders are fewer and report less remarkable differences in neuropsychological profiles, compared to those on adults. Lower premorbid IQ in developmental age has also been described as a risk factor for MDD (33). A study with Wechsler Intelligence Scale for Children (WISC) III and executive functions in children found only lower PSI and trial making test performance in patients with MDD (34).

Association between DMDD and lower school functioning is remarkable (35, 36), and this is partly accounted for by a high rate of comorbid learning disorders (LD) (37). Studies on neuropsychology of patients with SMD are apparently contradictory. In Adelman et al. (38) no differences were found in IQ between patients with SMD or bipolar disorder and controls, while in Rau et al. (39) SMD patients had slightly lower IQ at Wechsler abbreviated scale of intelligence. Recent literature has shown poorer motor inhibition, more developmental motor coordination disorder and written language disorder in DMDD than in psychiatric controls (37).

In a transdiagnostic approach, irritability has been shown to impact the association between inattention symptoms and difference in processing efficiency between conflict and non-conflict conditions in ADHD (40). This finding suggests DMDD patients may have a specific impairment when ADHD is also present. ADHD and LD are frequent comorbidities in adolescent depressive disorders, particularly in DMDD (20), and are known to influence intellective performance on Wechsler scales. Few studies have investigated cognitive abilities in mood disorders comorbid with ADHD and LD. In a study on adolescents with autism spectrum disorder, ADHD, or LD and comorbid anxiety, depression and behavior disorders, neurodevelopmental disorder modified significantly WISC III scores, while anxiety and affective comorbidities did not (41). In a more recent study on neuropsychology of neurodevelopmental and affective comorbid disorders in adults, however, depression exhibited a distinctive additive effect on ADHD patients, with more impaired processing speed, delayed recall of conceptual verbal information and shifting tasks (42).

Adaptive abilities and functioning show a grade of correlation with intelligence (43) but represent different constructs and hardly overlap. Performing adaptive abilities requires the integration of cognition, emotions, and behaviors to meet the demands of different environments (44). Tests of adaptive abilities measure abilities which are actually performed, a parameter which can be modified over time more than cognition (45). Functioning does not only relate to adaptive abilities performed, but is also affected by different maladaptive behaviors (46). Adaptive abilities are under-investigated in adolescents with mood disorders. General functioning in adolescents with different depressive and related disorders has been studied by some authors with mixed results (42).

We performed an exploratory study on cognitive, adaptive ability and general functioning in a cohort of adolescent inpatients with unipolar mood disorders, admitted in our psychiatry inpatients unit for acute decompensation, to better describe consistency of these different phenotypes. We aim to test two hypotheses:

1. Different unipolar disorders (MDD, DMDD and depressive disorder Not Otherwise specified DD-NOS) are characterized by distinctive impairment in cognitive and adaptive profile and functioning.
2. The diagnosis of mood disorder accounts for a difference in cognitive and adaptive profile and functioning among the different groups studied when controlling for neurodevelopmental and other psychopathological comorbidities.

## 2 Materials and methods

### 2.1 Population

We conducted a retrospective study on inpatients admitted from 2020 to 2022 to the Child Neuropsychiatry Unit of a referral center for children and adolescent psychiatry. We collected data on consecutive patients admitted for the first time, referred by either our own or regional emergency departments, secondary care (child psychiatrists in national healthcare centers) or our own outpatient service. We included children and adolescents of both sexes aged ≥11 to <18 years at admission with a diagnosis of DMDD, MDD or DD-NOS. We excluded patients with intellectual disability of mild to profound severity and/or full-scale IQ <70, patients with a diagnosis of (hypo)manic/mixed episode, patients with a diagnosis of substance-induced mood disorder and/or mood disorder due to another medical condition according to DSM-5 (6), patients with neurological comorbidities reported on history or diagnosed during hospitalization. Parents/legal guardians of each patient provided written, informed consent at clinic intake for potential research analysis and anonymous reporting of findings in aggregate form, in compliance with research ethics and with Italian legal and ethical requirements for clinical data. The study was approved by our institutional review board (Ethics Committee, Ospedale Pediatrico Bambino Gesù, Reference no. 2921/2022).

### 2.2 Measures of evaluation

Sociodemographic data were collected on clinical records on admission. Neuropsychological and psychopathology assessment was completed within 48 hours of admission. Diagnoses were assessed with the Kiddie-Schedule for Affective Disorders and Schizophrenia for School-aged Children, Present and Lifetime version (K-SADS-PL) (47), following DSM-5 criteria (6). Comorbidities were assessed with the same instrument of evaluation. Data on LD were collected by chart review, and confirmed only if structured standardized tests appropriate to scholarity had been performed and confirmed the presence of a LD. If subthreshold psychotic symptoms were detected or in case of clinical suspicion, SIPS/SOPS interview (48) was administered to investigate the presence of prodromal syndrome (ultra-high-risk state for psychosis, UHR), which has been further analyzed as a comorbid condition. The interview is composed of 19 items, each representing a different possible subthreshold psychotic symptom, yielding 4 constructs: positive, negative, disorganized, and general symptoms. Each item is rated on a 7-point scale (49) and the spectrum of high-risk states was defined as per current literature (50).

All diagnoses were reviewed before discharge by two independent expert clinicians (M.A. and R.A.) and agreement was reached in every included case.

Measures of cognitive profile were Wechsler intelligence batteries. WISC-IV (51) was used for patients aged 16 or under and Wechsler Adult Intelligence Scale (WAIS-IV) (52) was used for patients 17 years old or above. Both batteries comprised of 10 core subtests which combine to form four psychometrically validated factor scores: the Verbal Comprehension Index (VCI), the Perceptual Reasoning Index (PRI), the WMI and the PSI. All 10 subtests combine to form a full-scale IQ (FSIQ) score. FSIQ and composite scores of VCI, PRI, WMI and PSI were used for the analysis. Single subtests of the scale were always less discrepant than 5 points at an individual case level in included patients, so the four indexes were interpretable, supplemental subtests were not used. When the FSIQ was difficult to interpret because of a difference between the most discrepant indexes of 22 or more, a General Ability Index was calculated based on VCI and PRI alone and used as a measure of general intellect (53).

Adaptive abilities were assessed with the Adaptive Behavior Assessment System, Second Edition (ABAS-II) (54), which was administered to both parents/principal caregiver if parents were missing. It is a parent-report questionnaire which measures skills related to development, behavior, and cognitive abilities in 10 functioning areas, gathered in three main adaptive domains: conceptual (CAD), practical (PAD), social (SAD), and a comprehensive score, General Adaptive Composite (GAC), given by the sum of scaled scores from the functioning areas.

The Children’s Global Assessment Scale (C-GAS) (55) was used to assess global functioning.

### 2.3 Statistical analysis

Categorical variables are presented as number and percentage. Continuous variables are presented as mean and standard deviation (SD). In the section on the description of the cohort characteristics, association between variables was tested with Pearson’s or Fisher’s test, as appropriate. The Bonferroni correction for multiple comparisons was applied.

To test the 1^st^ hypothesis, means of cognitive, adaptive abilities and general functioning scores were compared in the different diagnosis groups by a multivariate analysis of variance (MANOVA). Mean IQ, Wechsler scale indexes (VCI, PRI, WMI, PSI), adaptive behavior general composite score and scales (Communication, Daily living skills, Socialization) and C-GAS score were compared between the 3 diagnosis groups. Variance–covariance homogeneity assumption was tested with Box’s test and was not violated. Pillai’s trace was chosen to assess the significance of the model. Eta squares (η^2^) were reported as a measure of effect size of the model. *Post-hoc* analyses were performed using Fisher’s LSD test. Odds ratios (OR) with 95% confidence intervals were reported. SPSS generalized linear model for repeated measures was used to test differences between VCI, PRI, WMI and PSI within patients with the same mood disorder diagnosis. VCI, PRI, WMI and PSI were computed as repeated measures and the three mood diagnosis disorder groups were tested independently.

To test the 2nd hypothesis, linear regression modeling was used to test the effect of mood disorder diagnosis controlling for potential confounding effect of age, sex and comorbidities known to potentially affect cognitive functioning, adaptive abilities and general functioning. Among these, we tested the variables which were found to be significantly different among mood disorder diagnoses in the previous analyses as predictors. FSIQ and indexes (VCI, PRI, WMI and PSI) were tested as predicted variables in repeated analyses. Furthermore, mood disorder diagnosis FSIQ and indexes, sex and comorbidities (if found to be significantly different among mood disorder diagnoses in previous analyses), were tested as predictors and ABAS-II general composite score and subscales as predicted variables. Lastly, mood disorder diagnoses, FSIQ and indexes, ABAS-II general composite score and subscales age, sex, comorbidities (if significantly different among mood disorder diagnoses in the previous analyses), were tested as predictors of C-GAS score.

A *p* value of 0.05 or less was considered significative. Analyses were conducted with Microsoft Office 365 – Excel and IBM SPSS Statistics V26 software.

## 3 Results

### 3.1 Characteristics of the cohort

During the study period, data on 198 patients (85.9% females) were collected. The diagnosis was of MDD in 33.3% (number = 66), DMDD in 6.6% (number = 13) and DD-NOS in 60.1% (number = 119). UHR for psychosis was present in 22.7% of patients. Patients had a mean 0.62 active comorbidities (SD 0.67, range 0–3). Female prevalence was lower in DMDD (61.5%, *p* = 0.012). DMDD patients had higher rates of ADHD (15.4%), LD (23.1%) and CD (7.7%) than other mood disorder groups and lower rates of current (*n* = 0) or past anxiety disorders (23.2%) or eating disorders (*n* = 0). Difference in prevalence of comorbid eating disorder was not significative after applying Bonferroni correction for multiple comparisons.

### 3.2 Hypothesis 1: cognitive and adaptive profiles and functioning differ according to mood disorder diagnosis

For a complete description of the cohort, see Table 1. Cognitive evaluation was carried out with WISC-IV in 84.3% cases and WAIS in the remaining cases. FSIQ was interpretable in 91.4%.

**Table 1:** Description of the cohort.

	MDD (n = 66)	DD-NOS (n = 119)	DMDD (n = 13)	Total (n = 198)	*p*
Age (years)	15.5 (±1.5)	15.5 (±1.3)	15.0 (±1.5)	15.5 (±1.4)	0.416
Female sex	92.4% (61)	84.9% (101)	61.5% (8)	85.9% (170)	0.012
Length of hospital stay (days)	5.4 (±3.3)	4.1 (±3.2)	4.4 (±2.0)	4.5 (±3.2)	0.028
History of anxiety disorder (any, lifetime)	65.2% (43)	74.8% (89)	23.2% (3)	68.2% (135)	0.001
UHR for psychosis	16.7% (11)	27.7% (33)	7.69% (1)	22.8% (45)	0.870
Comorbid anxiety disorder (any)	27.3% (18)	23.5% (28)	0	23.2% (46)	0.024
Comorbid panic disorder	0	5.88% (7)	0	3.54% (7)	0.090
Comorbid eating disorder (any)	24.2% (16)	13.4% (16)	0	16.2% (32)	0.042
Comorbid ADHD	1.51% (1)	2.52% (3)	15.4% (2)	3.03% (6)	0.025
Comorbid LD	3.03% (2)	5.90% (7)	23.1% (3)	6.06% (12)	0.021
Comorbid OCD	1.51% (1)	0.84% (1)	0	1.01% (2)	0.846
Comorbid CD	0	0	7.69% (1)	0.51% (1)	0.001
Comorbid ODD	1.51% (1)	2.52% (3)	0	2.02% (4)	0.777
Comorbid SUD	1.51% (1)	0.84% (1)	0	1.01% (2)	0.846
Comorbid PTSD	1.51% (1)	1.68% (2)	0	1.51% (3)	0.895
Number of present comorbidities	0.73 (±0.69)	0.55 (±0.66)	0.69 (±0.63)	0.62 (±0.67)	0.196
Cognitive profile					
FSIQ	105.2 (±13.8)	102.1 (±14.6)	87.2 (±10.7)	102.2 (±14.7)	<0.001*
VCI	109.3 (±14.2)	108.6 (±14.1)	96.6 (±11.9)	108.0 (±14.3)	0.010*
PRI	105.6 (±13.0)	105.5 (±14.8)	93.2 (±13.3)	104.7 (±14.4)	0.011*
WMI	95.9 (±16.3)	90.9 (±15.1)	81.7 (±9.3)	91.9 (±15.5)	0.005**
PSI	98.3 (±15.1)	93.8 (±15.4)	78.2 (±9.0)	94.3 (±15.7)	<0.001†
Adaptive profile					
ABAS II GAC	89.6 (±16.6)	86.9 (±19.3)	68.8 (±16.8)	86.6 (±18.9)	0.002*
ABAS II CAD	95.9 (±18.4)	91.9 (±18.3)	76.1 (±17.4)	92.19 (±18.7)	0.004*
ABAS II SAD	88.1 (±17.9)	83.2 (±18.4)	68.7 (±17.8)	83.9 (±18.7)	0.003*
ABAS II PAD	90.3 (±15.9)	87.8 (±18.6)	72.7 (±17.3)	87.7 (±18.1)	0.009*
Functioning					
C-GAS	47.2 (±6.0)	47.8 (±6.1)	45.9 (±5.2)	47.5 (±5.90)	0.503

*Significantly lower in DMDD than in MDD and DD-NOS.

**Significantly higher in MDD than in DMDD and DD-NOS.

†Significative differences in all pairwise comparisons. Nominal variables have been presented as % (number). Quantitative variables have been presented as mean (± standard deviation).

Abbreviations: MDD, mayor depressive disorder; DD-NOS, depressive disorder – not otherwise specified; DMDD, disruptive mood dysregulation disorder; UHR, ultra-high risk state for psychosis; ADHD, Attention Deficit Hyperactivity Disorder; LD, learning Disorder; OCD, obsessive compulsive Disorder; CD, conduct disorder; ODD, oppositional Defiant Disorder; SUD, substance Use Disorder; PTSD, post-traumatic stress disorder; FSIQ, full scale intelligence quotient; VCI, verbal comprehension index; PRI, perceptual reasoning index; WMI, working memory index; PSI, processing speed index; ABAS, Adaptive Behavior Assessment System; GAC, General Adaptive Composite score; CAD, Conceptual Adaptive Composite score; PAD, practical Adaptive Composite score; SAD, social Adaptive Composite score; C-GAS, Children’s Global Assessment Scale.

DMDD was characterized by a mean FSIQ of 87.8, which was significantly lower than MDD (105.2) and DD-NOS (102.1).

VCI and PRI were significantly lower in DMDD than in MDD and DD-NOS. WMI was significantly higher in MDD than in DD-NOS and DMDD, while in DMDD it was not significantly lower than in DD-NOS. Significant differences in PSI were found on post-hoc analysis in all comparisons between group, and PSI in MDD was significantly higher than in DD-NOS and in DMDD. Additionally, PSI in DD-NOS was significantly higher than in DMDD.

With regard to within-group difference between indexes, WMI and PSI were significantly lower than VCI and PRI in all diagnosis groups (*F* = 18.6, *p* < 0.001, partial *η^2^* = 0.47 in MDD group; *F* = 55.3, *p* < 0.001, partial *η^2^* = 0.59 in DD-NOS group; *F* = 6.5, *p* = 0.01, partial *η^2^* = 0.66 in DMDD group;). Pairwise comparisons of VCI vs. WMI, VCI vs. PSI, PRI vs. WMI, and PRI vs. PSI were significant with *p* ≤ 0.001 in MDD group and in DD-NOS group. In DD-NOS, VCI was also significantly higher than PRI (108.6 ± 14.1 vs. 105.5 ± 14.8, *p* = 0.023). In DMDD group, pairwise comparisons of VCI vs. WMI and VCI vs. PSI were significant with *p* = 0.001, pairwise comparison of PRI vs. WMI was significant with *p* = 0.002 and pairwise comparison of PRI vs. PSI was significant with *p* = 0.008.

With regard to adaptive abilities, patients with DMDD have lower mean General Adaptive Composite scores than MDD and DD-NOS; lower mean Conceptual Adaptive Domain scores than MDD and DD-NOS; lower mean Social Adaptive Domain scores than MDD and DD-NOS; and lower mean Practical Adaptive Domain scores than MDD and DD-NOS.

Global functioning measured with C-GAS was not significantly different among mood disorder diagnosis groups (*p* = 0.503).

For further details, please refer to Table 1.

### 3.3 Hypothesis 2: cognitive and adaptive profiles and functioning differ according to mood disorder diagnosis after controlling for comorbidities

We tested the effect of mood disorder diagnoses in predicting FSIQ and Wechsler scale indexes controlling for the effect of possible confounding variables which potentially influence cognitive indexes studied and significantly differ between groups of mood disorder diagnoses. In more detail, FSIQ and subsequently each index (VCI, PRI, WMI, and PSI) were set as predicted variables and mood disorder diagnosis, together with female sex, comorbid anxiety disorders, comorbid ADHD, comorbid LD and comorbid CD as predictors in five consecutive regression analyses. Results are shown in Table 2. A diagnosis of DMDD was a significant predictor of FSIQ, VCI, PRI, and PSI, correlating with lower scores even controlling for the effect of sex and comorbidities. However, comorbid LD was a significant predictor of FSIQ and of PRI, correlating with lower scores. PSI, in addition, was also significantly predicted by female sex and comorbid anxiety disorder, which were correlated with higher scores.

**Table 2:** Linear regression analyses for FSIQ, VCI, PRI, WMI, PSI.

Predicted variables	FSIQ *F* = 3.83; *p* = 0.001; r^2^ = 0.127		VCI *F* = 2.56 *p* = 0.040; r^2^ = 0.074		PRI *F* = 3.174 *p* = 0.003; r^2^ = 0.105		WMI *F* = 2.52 *p* = 0.017; r^2^ = 0.085		PSI *F* = 4.721 *p* < 0.001; r^2^ = 0.149	
	B (95% IC)	*p*	B (95% IC)	*p*	B (95% IC)	*p*	B (95% IC)	*p*	B (95% IC)	*p*
Intercept	98.14 (92.39–103.90)	<0.001	106.91 (101.16–112.65)	<0.001	103.07 (97.36–108.77)	<0.001	85.97 (79.75–92.18)	<0.001	87.46 (81.41–93.51)	<0.001
MDD	2.26 (−2.08–6.59)	0.306	0.23 (−4.06–4.53)	0.915	−0.64 (−4.91–3.62)	0.766	4.18 (−0.47–8.82)	0.078	3.60 (−0.92–8.12)	0.117
DMDD	**−11.21** (**−19.93**–−**2.49**)	**0.012**	**−10.13** (**−18.84**–−**1.41**)	**0.023**	**−8.94** (**−17.59**–−**0.29**)	**0.043**	−5.70 (−15.12–3.73)	0.235	**−10.56** (**−19.74**–−**1.39**)	**0.024**
Female sex	4.64 (−1.28–10.56)	0.124	2.01 (−3.89–7.92)	0.502	3.64 (−2.23–9.50)	0.223	6.20 (−0.19–12.58)	0.057	**6.55** (**0.34**–**12.77**)	**0.039**
Comorbid anxiety disorder	3.46 (−1.37–8.28)	0.159	2.36 (−2.37–7.10)	0.326	1.39 (−3.31–6.10)	0.559	1.48 (−3.65–6.60)	0.57	**5.62** (**0.63**–**10.60**)	**0.027**
Comorbid ADHD	−7.40 (−20.07–5.27)	0.251	−9.34 (−22.00–3.32)	0.147	−7.19 (−19.76–5.39)	0.261	−8.66 (−22.36–5.04)	0.214	−10.82 (−24.15–2.52)	0.111
Comorbid LD	**−8.98** (**−17.51**–−**0.45**)	**0.039**	−6.04 (−14.56–2.48)	0.164	**−13.81** (**−22.27**–−**5.35**)	**0.002**	−7.06 (−16.28–2.16)	0.132	−5.10 (−14.07–3.87)	0.263
Comorbid CD	8.46 (−22.84–39.77)	0.594	18.56 (−12.73–49.86)	0.243	15.06 (−16.02–46.13)	0.34	7.39 (−26.46–41.24)	0.667	1.92 (−31.03–34.88)	0.909

Linear regression analyses for FSIQ, VCI, PRI, WMI, PSI. Mood disorder diagnoses, sex, comorbid anxiety disorders, ADHD, LD, CD were tested predictors. F, significance (p) and r^2^ are reported for each model. B coefficients with their 95% confidence interval and significance (p) are reported for each predictor tested in each model. Abbreviations: MDD, mayor depressive disorder; DMDD, disruptive mood dysregulation disorder; ADHD, Attention Deficit Hyperactivity Disorder; LD, learning Disorder; CD, conduct disorder; FSIQ, full scale intelligence quotient; VCI, verbal comprehension index; PRI, perceptual reasoning index; WMI, working memory index; PSI, processing speed index.

Thereafter, we tested the effect of mood disorder diagnosis in predicting adaptive abilities scores, controlling for the effect of FSIQ and indexes, female sex, comorbid anxiety disorders, comorbid ADHD, comorbid LD and comorbid CD in four consecutive regression analyses. Results are shown in Table 3. DMDD diagnosis was a significant predictor of ABAS II GAC, VCI and ABAS II SAD, correlating with lower scores even controlling for the effect of IQ, sex and comorbidities. Furthermore, WMI was a significant predictor of ABAS II GAC, of ABAS II CAD, ABAS II SAD, of ABAS II PAD, with a positive correlation. The effect of comorbid ADHD was significant on ABAS II CAD, correlating with lower scores.

**Table 3:** Linear regression analyses for ABAS II GAC, CAD, SAD, PAD.

Predicted variables	ABAS II GAC *F* = 2.68; *p* = 0.002; r^2^ = 0.159		ABAS II CAD *F* = 3.24; *p* < 0.001; r^2^ = 0.185		ABAS II SAD *F* = 1.97; *p* = 0.030; r^2^ = 0.122		ABAS II PAD *F* = 2.12; *p* = 0.018; r^2^ = 0.130	
	B (95% IC)	*p*	B (95% IC)	*p*	B (95% IC)	*p*	B (95% IC)	*p*
Intercept	45.60 (14.02–77.19)	0.005	64.59 (33.66–95.52)	<0.001	56.57 (24.32–88.82)	0.001	50.41 (19.62–81.19)	0.001
MDD	1.03 (−4.80–6.85)	0.728	1.48 (−4.21–7.17)	0.609	3.93 (−2.02–9.87)	0.194	1.10 (−4.56–6.76)	0.702
DMDD	**−12.95** (**−24.96**–−**0.94**)	**0.035**	−9.85 (−21.61–1.91)	0.100	**−12.43** (**−24.68**–−**0.17**)	**0.047**	−9.24 (−20.94–2.46)	0.121
Female sex	−1.14 (−9.26–6.99)	0.783	−3.93 (−11.74–3.89)	0.322	−3.52 (−11.67–4.62)	0.394	−0.59 (−8.36–7.19)	0.881
Comorbid anxiety disorder	−0.62 (−6.97–5.73)	0.847	−1.32 (−7.50–4.86)	0.674	3.40 (−3.06–9.85)	0.3	−2.93 (−9.09–3.22)	0.348
Comorbid ADHD	−13.07 (−29.71–3.57)	0.123	**−17.88** (**−34.18**–−**1.59**)	**0.032**	−4.18 (−21.16–12.80)	0.628	−8.31 (−24.53–7.91)	0.313
Comorbid LD	5.90 (−5.53–17.33)	0.31	−2.20 (−13.39–9.00)	0.699	4.30 (−7.36–15.97)	0.467	0.66 (−10.48–11.80)	0.907
Comorbid CD	0.63 (−40.12–41.38)	0.976	5.20 (−34.70–45.10)	0.797	−1.86 (−43.44–39.72)	0.930	−6.93 (−46.64–32.77)	0.731
FSIQ	−0.05 (−0.62–0.53)	0.872	0.27 (−0.29–0.84)	0.341	−0.28 (−0.87–0.31)	0.349	−0.04 (−0.60–0.52)	0.893
VCI	0.01 (−0.35–0.37)	0.971	−0.08 (−0.43–0.28)	0.666	0.17 (−0.20–0.53)	0.377	−0.01 (−0.36–0.34)	0.959
PRI	0.12 (−0.19–0.44)	0.447	−0.18 (−0.49–0.13)	0.251	0.06 (−0.26–0.38)	0.7	0.09 (−0.22–0.39)	0.567
WMI	**0.29** (**0.06**–**0.51**)	**0.012**	**0.25** (**0.04**–**0.47**)	**0.023**	**0.31** (**0.08**–**0.53**)	**0.008**	**0.22** (**0.00**–**0.43**)	**0.048**
PSI	0.08 (−0.16–0.31)	0.525	0.08 (−0.15–0.31)	0.491	0.05 (−0.19–0.29)	0.691	0.15 (−0.08–0.38)	0.207

Linear regression analyses for ABAS II GAC, CAD, SAD, PAD. Mood disorder diagnoses, sex, comorbid anxiety disorders, ADHD, LD, CD, FSIQ and indexes were tested predictors. F, significance (p) and r^2^ are reported for each model. B coefficients with their 95% confidence interval and significance (p) are reported for each predictor tested in each model. Abbreviations: MDD, mayor depressive disorder; DMDD, disruptive mood dysregulation disorder; ABAS, Adaptive Behavior Assessment System; GAC, General Adaptive Composite score; CAD, Conceptual Adaptive Composite score; PAD, practical Adaptive Composite score; SAD, social Adaptive Composite score; ADHD, Attention Deficit Hyperactivity Disorder; LD, learning Disorder; CD, conduct disorder; FSIQ, full scale intelligence quotient; VCI, verbal comprehension index; PRI, perceptual reasoning index; WMI, working memory index; PSI, processing speed index.

Lastly, we tested the effect of mood disorder diagnosis in predicting general functioning, controlling for the effect of adaptive abilities, FSIQ and indexes, female sex, comorbid anxiety disorders, comorbid ADHD, comorbid LD and comorbid CD in a regression analyses. Results are shown in Table 4. Specific mood disorder diagnoses were not significant predictors of functioning, while comorbid anxiety disorders were weakly associated with higher C-GAS scores.

**Table 4:** Linear regression analyses for C-GAS.

Predicted variable	C-GAS *F* = 2.25; *p* = 0.006; r^2^ = 0.179	
	B (95% IC)	*p*
Intercept	27.85 (17.09–38.62)	<0.001
MDD	−1.56 (−3.44–0.31)	0.101
DMDD	1.21 (−2.66–5.08)	0.538
Female sex	0.50 (−2.10–3.09)	0.707
Comorbid anxiety disorder	**2.16** (**0.12**–**4.21**)	**0.038**
Comorbid ADHD	3.10 (−2.28–8.49)	0.257
Comorbid LD	−0.33 (−4.05–3.39)	0.862
Comorbid CD	−2.39 (−15.36–10.58)	0.716
FSIQ	−0.10 (−0.29–0.08)	0.282
VCI	0.07 (−0.04–0.19)	0.229
PRI	0.02 (−0.09–0.12)	0.741
WMI	0.05 (−0.02–0.12)	0.19
PSI	0.06 (−0.01–0.14)	0.11
ABAS II GAC	0.02 (−0.11–0.15)	0.742
ABAS II CAD	0.02 (−0.08–0.11)	0.723
ABAS II SAD	0.04 (−0.04–0.11)	0.305
ABAS II PAD	0.04 (−0.05–0.12)	0.422

Linear regression analyses for C-GAS. Mood disorder diagnoses, sex, comorbid anxiety disorders, ADHD, LD, CD, ABAS II GAC, CAD, SAD, PAD, FSIQ and indexes were tested predictors. F, significance (p) and r^2^ are reported for the model. B coefficients with their 95% confidence interval and significance (p) are reported for each predictor. Abbreviations: MDD, mayor depressive disorder; DMDD, disruptive mood dysregulation disorder; ABAS, Adaptive Behavior Assessment System; GAC, General Adaptive Composite score; CAD, Conceptual Adaptive Composite score; PAD, practical Adaptive Composite score; SAD, social Adaptive Composite score; ADHD, Attention Deficit Hyperactivity Disorder; LD, learning Disorder; CD, conduct disorder; FSIQ, full scale intelligence quotient; VCI, verbal comprehension index; PRI, perceptual reasoning index; WMI, working memory index, PSI, processing speed index.

## 4 Discussion

We analyzed a cohort of adolescents with depressive disorders to identify relevant differences in cognitive, adaptive profile and functioning between the different groups of mood disorder diagnoses. Participants were selected from an inpatient setting, where they were admitted for acute decompensation of current psychopathology. All participants were in an active phase of the psychiatric disorder and a degree of impairment was evident in all.

In patients with DMDD, general intelligence and adaptive abilities were significantly lower. Furthermore, patients with DMDD were characterized by a higher rate of neurodevelopmental comorbidities. In more detail, patients with DMDD had more comorbid ADHD and LD, as well as CD, and lower lifetime and present anxiety comorbidities. WMI and PSI were significantly lower than VCI and PRI in all patients, as a nonspecific signature of mood disorder.

In regression analyses, the effect of DMDD on lower IQ, VCI, PRI and PSI was significant; an effect of LD on IQ and PRI was significant too. The effect of DMDD on lower adaptive abilities was significant and more marked in the social adaptive domain, while ADHD had a significant effect on conceptual adaptive domain. Functioning was low on average in all groups, and not significantly affected by specific mood disorder diagnoses. It was slightly higher, however, in patients with anxiety comorbidities.

DMDD is a debated entity among depressive disorders, however our exploratory findings on cognition and adaptive impairment relative to psychopathological comorbidities, in the landscape of previous literature, may point toward its conceptualization as a distinct disorder, whose features cannot be exhaustively included in the mood disorder chapter. We contemplate a reconsideration of DMDD as a bridge entity between neurodevelopmental and mood disorders.

We assessed comorbidities systematically and analyzed their possible impact on cognition, adaptive abilities and functioning in regression analyses. This was particularly relevant for ADHD and LD, which are known to influence Wechsler scales examination scores. The effect of bidirectional interaction between neurodevelopmental and mood disorders on cognition is an under investigated issue, which is worth exploring in future studies. ADHD and LD affected cognitive profiles more than anxiety, depression, and behavior disorders in one study (41), while ADHD and depression showed specific and additive effects on PSI in another one (42). Our evidence indicates that a correlation of DMDD with lower cognitive and adaptive performances is a correlate of DMDD itself and not only explained by associated neurodevelopmental comorbidities. This is also consistent with the findings of Haller et al. (40), who recently described significant interactive effects between ADHD and DMDD in cognitive processing and a pivotal role for irritability in processing efficiency between conflict and non-conflict conditions. This is also consistent with the recent description of Benarous et al. (37) of DMDD as an admixture of mood and developmental disturbances which is not explained by its association with ADHD. Patients with DMDD in our study can be described as children with chronic impairing irritability, with a mild grade of cognitive impairment and frequent overlapping neurodevelopmental disorders, with remarkably lower social abilities. On this basis, we describe DMDD as a distinct entity, straddling neurodevelopmental and mood disorders.

DMDD in our cohort appears distinct from pediatric bipolar disorders (10, 56) since studies on high-risk cohorts for bipolar disorder found a premorbid social and cognitive functioning comparable to the general population and more internalizing comorbidities than neurodevelopmental disorders (16). It also appears distinct from ADHD for the grade and quality of impairment. Some authors argue that DMDD may be conceptualized as a subtype of ADHD characterized by temper and mood lability (16, 57). Our patients with DMDD, however, have an impairment which encompasses all domains of cognition and affects skills for relation with others, while an additive effect of ADHD on mood disorder is more apparent in conceptual domain. Both conceptual and social adaptive domains affect academic functioning, but social skills may be viewed as a more general requisite for becoming independent in the context of a community and developing identity in adolescence (58). Language and communication give access to self-regulatory strategies and provide alternatives to disruptive outbursts, a maladaptive coping strategy which is a hallmark of DMDD (59). Recent studies (60) have investigated a specific neuropsychologic background which may underlie development of DMDD, characterized by deficits in emotion recognition, which may limit social adaptation and self-regulation. Neurobiology models of dysfunction of reward circuitry and learning in severe chronic irritability support this view. Brotman et al. (56) described a translational model, in which subjects with severe irritability are characterized by abnormal threshold and reactivity to frustration, with diffuse alterations in prefrontal and anterior cingulate cortex, striatum and amygdala. As noted by Benarous et al. (61), failure of children with DMDD to develop effective emotional regulation skills is rooted in early development and involves impairments in instrumental learning and in performing and learning from parent–child interactions.

These considerations are also interesting in light of recent studies on “emotional dysregulation” construct, conceptualized as a distinct neurodevelopmental, early-onset disorder (62). DMDD may be viewed as a neurodevelopmental early-onset disorder, where regulation of emotions is particularly affected on a cognitive deficiency basis, which reinforces social maladaptation over time. It would be worth exploring in further studies the possible construct overlap of emotional dysregulation, studied with specifically designed instruments, such as RIPoSt-Y (62), with DMDD and ADHD.

Our patients with DMDD had higher rates of ADHD, LD and CD, which is consistent with the studies of Benarous et al. (20, 37), but also lower rates of anxiety disorders. This may be due to the fact that we observed young patients, who are in the early phase of their psychopathological trajectory, a phase in which anxiety comorbidities may be less prevalent, while it is known that anxiety is relevant in longitudinal follow-up studies of chronically irritable children (63).

We also analyzed MDD, DD-NOS and DMDD to find differences in cognitive performance in different domains within each group to identify distinctive profiles. Impairment in working memory and processing speed appeared as a non-specific correlate of current mood disorder across all diagnoses. Literature on adults (28, 64, 65) and more sparse literature on adolescents (34) supports our finding of WMI and PSI being lower than VCI and PRI in mood disorders. WMI and PSI are critical during development for the acquisition of new learning and actual application of crystallized knowledge. WMI significantly correlated with adaptive abilities, accordingly. Of note, DD-NOS in our cohort showed a grade of cognitive and adaptive impairment comparable with MDD. A reflection on this significant impairment in depressed adolescents not meeting MDD criteria is needed, and we remark that these pictures may be impairing during the age of development and must not be overlooked. However, for a correct interpretation of this finding, we must comment on the intrinsic limit posed by the heterogeneity of DD-NOS diagnosis, characterized by definition by depressive symptoms which do not meet severity and duration criteria for MDD. DD-NOS are also often characterized by emotional dysregulation and mood instability which do not meet criteria for a bipolar disorder or for a DMDD diagnosis. A significant lowering of WMI in this group may be implied by further psychopathology which could have been observed only in a longitudinal study. Of note, our inpatients with DD-NOS also frequently met criteria for UHR for psychosis (28%).

PSI was the most discriminant index between groups. It was higher in females, consistently with literature (66), and since our sample is made predominantly of female patients, interpretation of this finding has some limitations. PSI links causally to other elements of intelligence (67, 68), permitting more effective use of WM and enhancing fluid reasoning (69, 70). Its impairment has been observed in euthymic adults with a previous diagnosis of MDD (64) and first-degree relatives of patients with mood disorders (29). Our results, in the wake of the cited literature, highlight its relevance in early-onset psychopathology. PRI and VCI seem to be relatively preserved in MDD and DD-NOS, with mean scores around 100. A lower PRI, however, is a correlate of LD, consistently with previous literature on visual–spatial and motion perception impairment in developmental dyslexia (71).

General functioning in our study was low on average in all groups, due to the inpatient setting of our study, where patients are admitted for acute decompensation of their psychopathology. Previous studies report mixed findings on functioning. A study on depressive disorders did not find differences in global functioning but reported more school and peer-relationship difficulties in DMDD compared to MDD (20). A study on inpatients found higher discharge Global Assessment of Functioning (GAF) scores in patients with DMDD (72), while another found lower GAF scores on admission and discharge (57).

A strength of our study is providing data on cognitive and adaptive profile of DMDD, whereas literature is limited. The evaluation procedure was standardized, providing a synoptic overview on cognition, adaptation and functioning, and all patients were evaluated in an early and acute phase of their psychopathological trajectory. There are, however, considerable limitations. Firstly, the study was exploratory, DMDD patients were a minority, and the sample size was underpowered. Chronic irritability is more often managed in outpatient units, limiting the number of our observations of DMDD to patients needing prompt start of a pharmacological treatment. Furthermore, due to the cross-sectional design of the study, some patients may have been provisionally classified as DD-NOS for not meeting the criteria for a diagnosis of another mood disorder (such as Persistent depressive disorder, or bipolar and related disorders, or even DMDD) at first clinical observation. Excessive prevalence of females in our cohort is consistent with differential incidence of mood disorders between sexes but limits the detection of sex differences in the outcomes studied. Finally, our cohort is made of inpatients, which are all characterized by higher severity of current psychopathology. These limitations are worth addressing in specifically designed perspective studies.

## Data availability statement

The datasets presented in this article are not readily available because of the nature of the research, due to ethical reasons. Further inquiries can be directed to the corresponding author.

## Ethics statement

The studies involving humans were approved by Ospedale Pediatrico Bambino Gesù. The studies were conducted in accordance with the local legislation and institutional requirements. Written informed consent for participation in this study was provided by the participants’ legal guardians/next of kin.

## Author contributions

MA: Conceptualization, Formal analysis, Writing – original draft, Writing – review & editing. MCP: Data curation, Methodology, Writing – review & editing. RA: Conceptualization, Investigation, Methodology, Writing – review & editing. ML: Data curation, Investigation, Writing – review & editing. MP: Methodology, Supervision, Writing – review & editing. SV: Conceptualization, Investigation, Supervision, Writing – review & editing.
